# An Insight into the Anticancer Activities of Ru(II)-Based Metallocompounds Using Docking Methods

**DOI:** 10.3390/molecules180910829

**Published:** 2013-09-04

**Authors:** Adebayo A. Adeniyi, Peter A. Ajibade

**Affiliations:** Department of Chemistry, University of Fort Hare, Private Bag X1314, Alice 5700, South Africa

**Keywords:** ruthenium complexes, anticancer, docking methods, receptors

## Abstract

Unlike organic molecules, reports on docking of metal complexes are very few; mainly due to the inadequacy of force fields in docking packages to appropriately characterize the metal atoms that consequentially hinder the rational design of metal-based drug complexes. In this study we have made used Molegro and Autodock to predict the anticancer activities of selected Ru(II) complexes against twelve anticancer targets. We observed that introducing the quantum calculated atomic charges of the optimized geometries significantly improved the docking predictions of these anticancer metallocompounds. Despite several limitations in the docking of metal-based complexes, we obtained results that are highly correlated with the available experimental results. Most of our newly proposed metallocompounds are found theoretically to be better anticancer metallocompounds than all the experimentally proposed RAPTA complexes. An interesting features of a strong interactions of new modeled of metallocompounds against the two base edges of DNA strands suggest similar mechanisms of anticancer activities similar to that of cisplatin. There is possibility of covalent bonding between the metal center of the metallocompounds and the residues of the receptors DNA-1, DNA-2, HDAC7, HIS and RNR. However, the general results suggest the possibility of metals positioning the coordinated ligands in the right position for optimal receptor interactions and synergistic effects, rather than forming covalent bonds.

## 1. Introduction

There have been several ruthenium-based complexes synthesized as anticancer drugs [1,2,3] to serve as alternative to cisplatin, which is the most widely used and efficient anticancer agent [1,4]. One of the major limitations in the rational design of organometallic anticancer complexes is the lack of proper knowledge about their anticancer targets [5,6,7,8,9]. In our research group we have been making efforts to predict the possible targets of metal-based complexes as anticancer agents using theoretical docking methods. However, unlike their organic counterparts, there is a very serious limitation in making use of docking tools for metal complexes, mainly due to lack of proper force fields to accommodate the metal centre [10]. Even in the quantum computation field, the volume of works on metal-based complexes are very low compared to organic compounds due to the high computational demand and difficulties of finding an appropriate method for their optimization [11].

In our previous work [12,13], we have made used of docking packages like Glide, Gold and Autodock whereby some interesting results were obtained. In this work we have made use of another docking package called Molegro, which recognizes the ruthenium atom as a metal centre better than Glide, Gold and Autodock. Also, we have enhanced its performance including that of Autodock by introducing the atomic charges of each metallocompound obtained from the quantum optimized structures of the compounds. Other new metallocompounds are designed which we have never reported before and the binding of co-crystallized compounds that accompanied each of the receptors is set as references in this computational docking. In addition to the number of receptors considered in our previous works [12,13], we have also included DNA as one of the targets to see the possibility of some of these metallocompounds having favourable interaction properties like that of cisplatin. Other receptors considered in these work besides DNA are thioredoxin reductase (TrxR) [14], histone protein in a nucleosome core particle (HP-NCP) [15], BRAF Kinase [16], recombinant human albumin (rHA) [17,18], thymidylate synthase (TS) [19], ribonucleotide reductase (RNR) [20], histone deacetylase (HDAC7) [20], cathepsin B (CatB) [14], topoisomerase II (TopII) [21,22] and DNA gyrase. All these have been reported to play significant roles in cancer growth and metastasis, except DNA gyrase, which is a notable bacterial enzyme [23,24], considered in this project for the purpose of seeing if some of these metallocompounds can play a dual role.

## 2. Results and Discussion

### 2.1. General Features of the Binding Activities of the Metallocompounds

**Figure 1:**
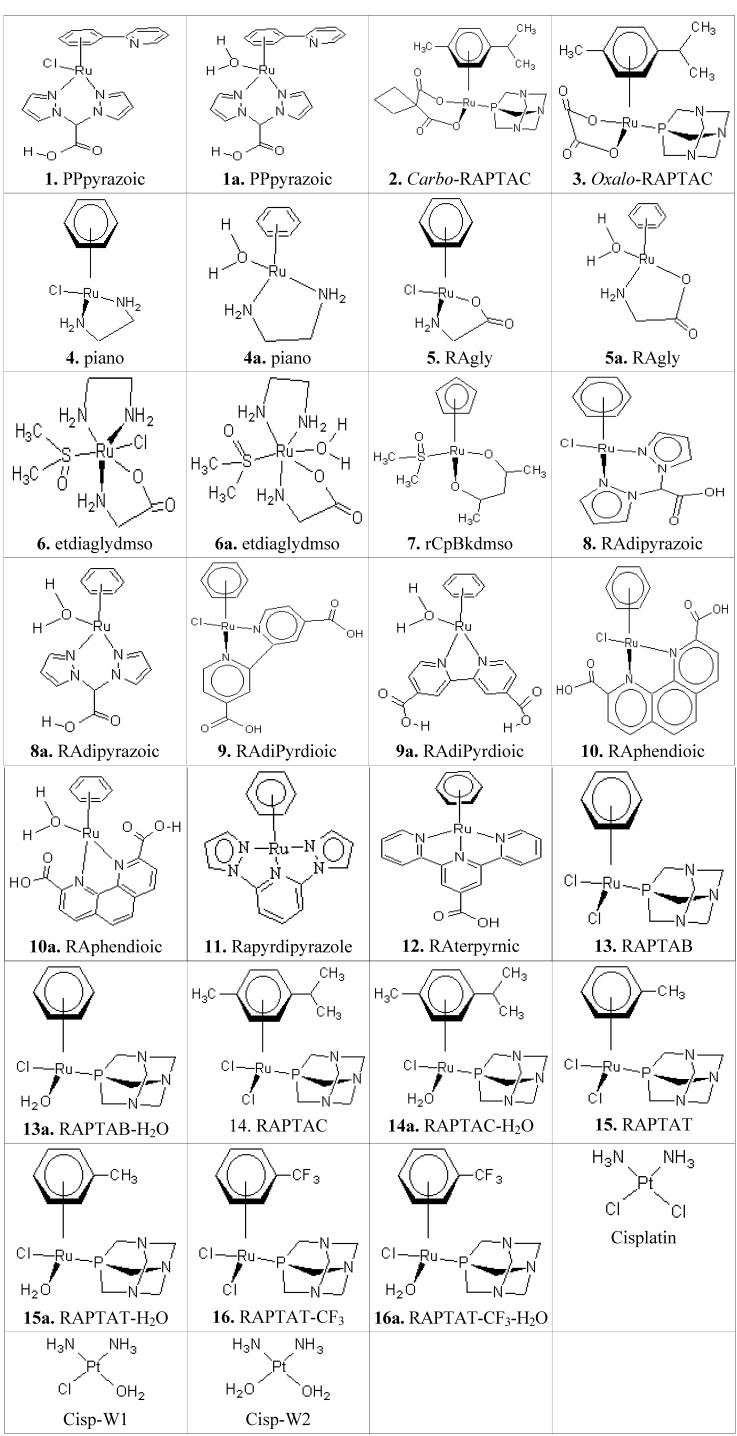
The schematic structures of the anticancer metal-based complexes.

In this paper the binding affinities and conformations of 30 metallocompounds including their hydrated forms (Figure 1) are reported. The 30 metallocompounds are docked against twelve receptors using the relaxed Molegro, constrained Molegro and Autodock docking approaches. The summary of 72 poses of the best two metallocompounds in their interaction with the receptors are shown in Table 1, indicating the hydrogen bonding (HB) and the possible metal-receptor (MR) interactions with the receptor binding site residues. The results obtained from the docking of metallocompounds to twelve receptors using Autodock, relaxed Molegro without metal-residue covalent constraints and constrained Molegro with covalent constraints are shown in Table 2, Table 3, Table 4, Table 5, Table 6, Table 7, respectively. The docking results obtained from both relaxed and constrained Molegro docking (Table 4, Table 5, Table 6 and Table 7) give a better ranking than the Autodock which cannot not be easily ranked since many of the Autodock values (Table 2 and Table 3) are within its standard error margin of ~2.177 kcal/mol [25,26]. Table 2, Table 4 and Table 6 contain binding affinities of non-hydrated complexes, while Table 3, Table 5 and Table 7 contain the affinities of their respective hydrated complexes. The hydrated complexes are named after their respective non-hydrated type with the suffix “a”.

The PP = 4*-*Phenylpyridine, RA = Ruthenium-(η6-arene); etdia = ethylenediamine, phen = phenanthroline; pyr = pyridine; pyraz = pyrazole, pta = 1,3,5-triaza-7-phosphaadamantane; Cp = cyclopentadienyl, Bk = β-diketone, piano = model of piano-stool.

Generally, we observed that some of our new metallocompounds like complexes **1**, **1a**, **8**, **8a**, **9**, **9a**, **10**, **10a**, **11** and **12** (Table 4 and Table 5) have better binding affinities than the co-crystallized compounds of the CatB, HDAC7, TS and BRAF Kinase receptors. Though co-crystallized compounds bind better in TrXR, TopII, RNR and Gyrase than all the metallocompounds (Table 4, Table 5, Table 6 and Table 7), many of our modeled metallocompounds (occasionally the bidentate RAPTA complexes **2** and **3**) are found to be highly competitive with the co-crystallized compounds in their binding affinities (Table 4, Table 5, Table 6 and Table 7). The newly modeled metallocompounds, especially **1** and **1a**, **9** and **9a**, **10** and **10a**, **11** and **12** followed by the bidentate RAPTA (**2** and **3**) and hydrated RAPTA (**13a**, **14a**, **15a** and **16a**) complexes are predicted to have stronger binding affinity to many of the receptors (Table 4, Table 5, Table 6 and Table 7).

The proposed mechanism of activation of the metallocompounds through hydration [2,18,27,28] is further confirmed through the docking results. In most of the receptor interactions, Autodock indicates an enhanced binding energy with receptors for all the hydrated complexes (Table 4 and Table 5). The same thing is observed for the covalent constrained docking of metallocompounds to DNA-1,DNA-2, CatB, RNR and TrXR and also for relaxed Molegro docking to DNA-1 and DNA-2 (Table 2, Table 3, Table 4 and Table 5).In other receptors, only a few hydrated metallocompounds increase their binding energy as a result of the hydration and this depends on the type of the receptor targets. The binding activities of the hydrated forms of RAPTA complexes were greatly enhanced. Also, the bidentate form of RAPTA complexes are suggested to competitively bind strongly to receptors as the hydrated forms of RAPTA complexes, especially metallocompound **14a**, though their activities are still lower than the modeled metallocompounds using the results of the three methods of docking (Table 2 and Table 3; Table 4 and Table 5; Table 6 and Table 7). In many of the receptor interactions, metallocompounds **16** and **16a** are predicted to have better binding affinity than **15** and **15a** (Table 4 and Table 5) except for receptors RNR which further supports the experimental report of higher anticancer activities of the first pair [27].

In many instances, the binding of the relaxed Molegro docking are stronger than the constrained Molegro docking, also the relaxed Molegro agrees better with the available experimental reports (Table 8 and Table 9) and the experimentally proposed activation of metallocompounds by hydration (Table 4, Table 5, Table 6 and Table 7). This is an indication that the metal center will give preference to holding the coordinated ligands in appropriate position for optimal receptor residues interaction and synergistic effect than forming covalent bonding. In the absence of covalent constraints, the features of the affinities of the metallocompounds to receptors depends greatly on the type of metallocompounds from the results of strong binding interactions of **1** and **1a**, **8** and **8a**, **9** and **9a**, **10** and **10a**, **11** and **12** to most of the receptors. Even though the ranking of the metallocompounds against the receptors in the covalent constrained docking is similar to that of the unconstrained (Table 4, Table 5, Table 6 and Table 7) type, yet the features of the metallocompounds’ affinity depend greatly on the type of receptors.

All the docking methods clearly indicate that the best target of the hydrated cisplatin (Cisp-W1 and Cisp-W2) is DNA as they are found having higher binding energy with DNA compare to other receptors. The hydrated cisplatin, especially the doubly hydrated form (Cisp-W2) has the highest DNA binding compared to the other forms of cisplatin which further confirmed the need for hydration before activation [3,28]. However, in protein receptors, there is decrease in binding affinity of hydrated cisplatin compare to its non-hydrated type that further confirms that the protein is not its target. Considering the ranking of both relaxed and constrained Molegro docking, the best targets of the metallocomplexes follow the order HDAC7 > DNA-1 > rHA > CatB > DNA-2 > Gyrase > TrXR > TopII > TS > RNR > HiS > Kinase except in few cases like complex **12** that prefers CatB to rHA. The poorest binding affinity of the complexes is observed in the covalent constrained docking to rHA receptor. This suggest that the interaction of the metallocompounds with rHA as a transport receptor that is responsible for the pharmacokinetic availability of a wide range of drugs, including metallodrugs and consequentially determine their bioavailability and toxicology [29] is better described in terms of other interacting forces rather than covalent interaction of ruthenium metal with its residues.

### 2.2. The Interacting Poses of the Best Two Metallocompounds in the Receptor Binding Sites

**Figure 2:**
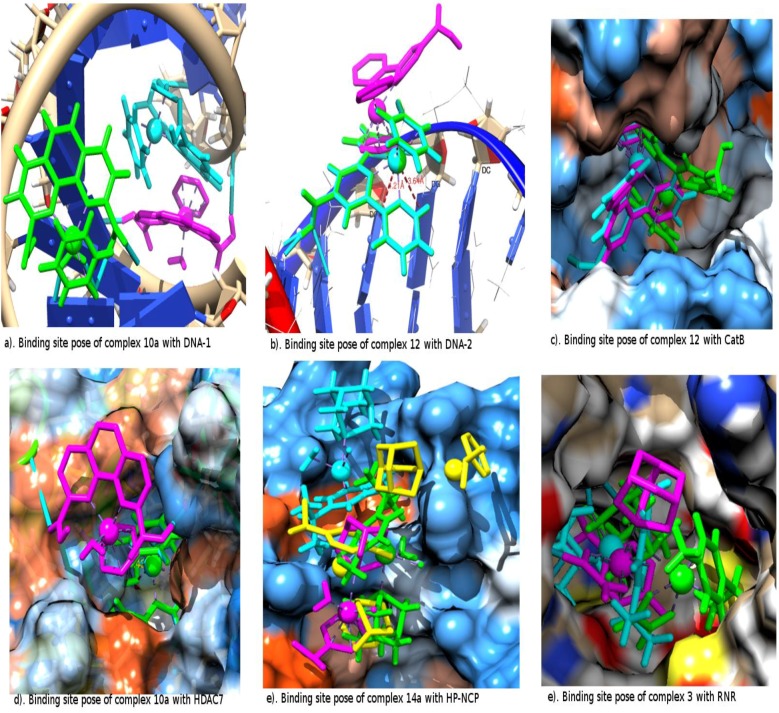
The receptor binding site interaction of the selected best binding metallocompounds from Autodock (magenta), unconstrained Molegro (cyan), constrained (green) and the experimental crystal structures (yellow).

To have a better understanding of the binding site orientation poses and interaction of the metallocompounds with the residues of each receptor, we have selected two metallocompounds predicted by the relaxed Molegro, covalent constrained Molegro and Autodock methods to have the best binding to each of the receptors (Table 1). The binding site interactions are elaborated in terms of the hydrogen bonds (HB) and the metal-residues (MR). The MR considers possible covalent bonding interactions of the metal with a nucleophilic Centre of receptor residue, which was considered during the constrained docking. There is a possibility of metal-residue (MR) interaction of complex 4a with DNA-1 according to Autodock prediction (Table 1). The relaxed Molegro and Autodock dockings suggest a different binding site from that of constrained docking in the binding of the metallocompound to DNA-1 (Figure 2a). However, in DNA-2 both relaxed and Autodock dockings suggest similar binding sites to that of covalent constrained docking for many of the metallocompound interactions. Interestingly, the suggested conformation of complex **12** binding to DNA-2 from both the relaxed and covalent constrained dockings is perfectly superimposed (Figure 2b). There is also an interesting feature of possible intercalation of complex **12** with the two based pair nitrogen atoms (N7) of the guanine in DNA-2 by both relaxed and constrained docking as shown in Figure 2b. Ranked among the best binding metallocompounds against the DNA-1 and DNA-2 are complexes **10a** and **12** by both the relaxed and covalent constrained docking. Autodock also predicted complex **10a** among the best five inhibitors of DNA within the interval of 0.60 Kcal/mol. The features of complex **10a** in Autodock docking to CatB shows a very strong H-bond interaction of one of its carboxylic groups with the SG of the CYS 29, which supposed to be, center of metal-residue covalent interaction (Table 1).

When covalent constraints were applied on the metallocompounds’ interaction with CatB, the possibility of metallocompound **12** forming HB with the receptor was traded for the formation of covalent bonds (Table 1) which subsequently led to a lower activity (comparing metallocompound **12** in Table 4, Table 5, Table 6 and Table 7). The same thing was observed for the interaction of metallocompound **1** with the TrXR receptor. Also, the tridentate ligand part of metallocompound **12** in the relaxed Molegro docking (cyan) and Autodock (magenta) fits properly into the hydrophobic pocket of the CatB as shown in Figure 2c, which was not the case in the constrained docking (green). This further suggest the possible reason why the binding affinities of the relax docking are higher than that of the constrained docking. The three methods of docking predicted **12** as one of the best binding metallocompounds to CatB. Also, the orientation and the interactions suggested Autodock and relaxed Molegro docking are the same with the structure of **12** on the binding site almost superimposed (Figure 2c).

Both relaxed and constrained Molegro docking show a superimposed conformation for complex **10a** in which the arene unit is pointing toward the mouth of the inner pocket of the HDAC7 while the Autodock preferentially locates its outer pocket (Figure 2d). Complex **1a** is rated to have the best binding affinity to HP-NCP in relaxed Molegro docking but it has no visible H-bond or metal-receptor interaction. The same is observed for Autodock docking of complex **5a** to BRAF Kinase and relaxed Molegro docking of complex **10** to TopII. These suggest the significant importance of other interacting forces like Van der Waal and close contact interactions. The ruthenium metal appears to have interaction with the oxygen atom of residue GLU 61G of the HP-NCP from the Autodock prediction and constrained docking. The conformation of complex **1a** as suggested by the relaxed Molegro docking fits in totally into one side of the binding site pockets than the conformation suggested by the constrained and Autodock docking to HP-NCP. In Figure 2e, the experimental conformation from the crystal structure of RAPTA complex **14a** in the HP-NCP binding site is compared to the conformation obtained from the docking results. This complex was rated best among the RAPTA complexes by Autodock; it is the fourth of the RAPTA complexes from relaxed Molegro while it is ranked low by constrained Molegro. However, the orientation suggested by the covalent constrained docking agrees strongly with one of the experimental orientations as shown in Figure 2e. Also the Autodock locates the same binding sites in agreement with the crystal structure but associated with the reverse orientation of complex **14a** while relaxed Molgro located only one of the pockets suggested by the crystal structure.

In Autodock docking of complex **10** to rHA we observed possible covalent interactions of ruthenium with the NH_3_ unit of the LYS 195A residue characterized by a distance of 1.78 Å (Table 1). The three methods of docking suggested either the hydrated complex **10a** or non-hydrated complex **10** as the best binding metallocompound to rHA. They all predicted complex **1** as the best for RNR. The suggested conformation for the non-hydrated and hydrated complexes **1** and **1a**, respectively, by constrained docking in RNR is almost superimposed with a little variation in the orientation of their arene unit. The conformation suggested for the docking of RAPTA complex **3** to RNR is shown in Figure 2f; both Autodock and Molegro suggest a very close position for the ruthenium atom but a different position for its coordinated ligands. In the constrained docking of complexes **9a** and **8a** to TrXR, there is possibility of the ruthenium atom forming a covalent bond with the bridged surphur (SG) residue CYS 64A or strong close contact van der Waal interactions with the two bridged surphur (SG) atoms of the residues CYS 64A and CYS 59A.

**Table 1:** The two best binding metallocomponds of each receptor from Molegro and Autodock showing the existing Hydrogen Bond (HB) (in Å) and Metal-Residues (MR) interactions with binding site residues.

DNA-1	Molegro	metallocompound **10a** {[HB: 2.39 O(COOH) ^i^ H(DC 18.B)], [HB: 1.91 H(COOH) ^ii^ N7(DG 16.B)]};
		metallocompound **12** {[HB: 2.32 O(COOH) H(DA 17.B)], [HB: 1.44 H(COOH) O6(DG 7.A)]}
	Molegro-Constrained	metallocompound **10a** {[HB: 1.71 O(COOH) ^i^ H(DC 2.A)], [HB: 1.89 H(COOH) ^i^ O6(DG 23.B)], [HB: 1.66 H(H_2_O) O6(DG 23.B)], [HB: 2.01 H(H_2_O) N7(DG 23.B)], [MR 4.49 N7(DG 34.B)], [MR 4.60 N7(DA 22.B)]};
		metallocompound **9a** {[HB: 2.05 H(COOH) ^i^ O6(DG 23.B)], [HB: 2.02 H(COOH) ^ii^OP2(DG 23.B)], [HB: 2.11 H(H_2_O) N7(DA 22.B)], [MR 4.60 N7(DA 22.B)]};
	Autodock	metallocompound **9a** {[HB: 2.00 H(COOH) ^i^ OP1(DT 5.A)], [HB: 1.65 H(COOH) ^ii^ OP2(DA 15.B)]}
		metallocompound **4a** {[HB: 1.81 H(NH_2_) ^i^ OP1(DG 6.A)], [HB: 1.70 H(NH_2_)^ii^ OP2(DG 7.A)], [MR 3.32 O(DG 6.A)], [MR 3.88 O(DG 6.A)]}
DNA-2	Molegro	Metallocompound **10a** {[HB: 1.92 O(COOH) ^i^ H(DG 7.T)], [HB: 1.76 H(COOH) ^i^ O4(DT 8.T)], [HB: 2.16 H(COOH) ^ii^ O3(DC 9.T)]};
		metallocompound **1a** {[HB: 1.75 H(COOH) O2(DC 8.P)], [HB: 2.12 H(H_2_O) O4(DT 8.T)]}
	Molegro-Constrained	metallocompound **12** {[HB: 1.57 H(COOH) O6(DG 5.P)], [HB: 2.50 O(COOH) H(DC 9.T)], [HB: 2.80 O(COOH) H(DA 6.P)], [MR 3.64 N7(DG 6.T)], [MR 4.21 N7(DG 7.T)]};
		metallocompound **1** {[HB: 1.95 N(arene) H(DA 6.P)], [MR 3.91 N7(DG 7.T)]}
	Autodock	metallocompound **1a** {[HB: 1.79 H(COOH) OP2(DG 6.T)]};
		metallocompound **4a** {[HB: 1.76 H(H_2_O) OP2(DG 7.T)], [HB: 1.96 H(NH_2_) ^i^ O3(DG 6.T)], [HB: 1.84 H(NH_2_) ^i^ OP1(DG 6.T)]}
CatB	Molegro	metallocompound **12** {[HB: 1.66 H(COOH) O(GLY 24 D)]}
		metallocompound **1** {[HB: 2.21 H(COOH) O(MET 196 E)]}
	Molegro-Constrained	metallocompound **1a** {[HB: 3.28 N(arene) SG(CYS 29D)], [HB: 2.61 H(COOH) O(GLY 198E)], [MR 4.60 SG(CYS 29D)]}
		metallocompound **8a** {[MR 4.60 SG(CYS 29D)]}
	Autodock	metallocompound **10a** {[HB: 2.99 O(COOH) ^i^ SG(CYS 29 D)], [HB: 2.76 O(COOH) ^i^ H(GLN 23 D)], [HB: 1.70 H(COOH)^i^ OE1(GLN 23D)], [HB: 2.14 H(COOH) ^ii^ OE2(GLU 122D)]};
		metallocompound **1a** {[HB: 1.72 H(COOH) OE1(GLU 122 E)], HB: 1.72 H(COOH) OE2(GLU 122E)]}
Gyrase	Molegro	metallocompound **10** {[HB: 2.17 H(COOH) O(ASP 73A)]}
		metallocompound **11** {[none]}
	Molegro-Constrained	metallocompound **10a** {[HB: 2.24 H(COOH) ^i^ OD2(ASP 49A)], [HB: 1.92 H(COOH) ^ii^ O(GLY 117A)], MR: 4.60 ND2(ASP 46 A)]}
		metallocompound **9a** {[HB: 1.50 H(H_2_O) OD1(ASP 46A)], [HB: 2.08 H(COOH) ^i^ O(ASP 46A)], [HB: 1.84 H(COOH) ^ii^ O(ASP 45A)], MR: 4.27 ND2(ASP 46 A)]};
	Autodock	metallocompound **12** {[HB: 2.11 H(COOH) O(ASP 73A)], [HB: 1.81 H(COOH) O(ASP 73A)], [HB: 2.00 O(COOH) H(GLY 77A)]};
		metallocompound **9a** {[HB: 1.90 O(H_2_O) HD22(ASN 46A)], [HB: 1.63 H(COOH) ^i^ O(LYS 103 A)], [HB: 2.05 O(COOH) ^ii^ H(ALA 100 A)], [HB: 1.65 H(COOH) ^ii^ O(ILE 94 A)], [HB: 2.14 O(COOH) ^ii^ H(SER 121 A)]}
HDAC7	Molegro	metallocompound **1** {[HB: 1.82 N(arene) H(imi@HIS 709A)], [HB: 1.87 O(COOH) H(imi@HIS 669A)]};
		metallocompound **12** {[HB: 2.36 H(COOH) O(ASP 707A)]}
	Molegro-Constrained	metallocompound **10a** {[HB: 1.89 H(COOH) ^i^ O(GLY 678A)], [HB: 2.43 O(COOH) ^i^ H(CYS 680A)], [HB: 1.99 O(COOH) ^i^ HE2(HIS 669A)], [MR: 3.43 NE2(HIS 709A)]}
		metallocompound **11** {[MR: 3.60 N(HIS 709A)]}
	Autodock	metallocompound **1a** {[HB: 1.63 H(COOH) OD1(ASP 626A)], [HB: 1.90 H(H_2_O) OD1(ASP 626A)]};
		metallocompound **9a** {[HB: 1.80 O(COOH)^i^ H(PHE 738A)], [HB: 1.79 H(COOH) ^i^ O(PRO 809 A)]}
HP-NCP	Molegro	metallocompound **1a** {[none]}
		metallocompound **11** {[MR: 3.38 OE(GLU 56G)]}
	Molegro-Constrained	metallocompound **10a** {[HB: 1.75 H(COOH) ^i^ OE1(GLU 92 G)], [[HB: 2.17 H(COOH) ^ii^ NE2(HIS 106 H)], [MR: 4.36 OE1(GLU 61 H)], MR: 4.44 NE2(HIS 106 H)]}
		metallocompound **11** {[MR: 4.36 OE1(GLU 61 H)], [MR: 4.60 NE2(HIS 106 H)]}
	Autodock	metallocompound **4a** {[HB: 1.67 H(H_2_O) OE2(GLU 61 G)], [HB: 1.67 H(H_2_O) OE1(GLU 64G)]};
		metallocompound **5a** {[MR: 2.90 OE2(GLU 61 G)]}
BRAF Kinase	Molegro	metallocompound **1a** {[HB: 2.09 H(COOH) O(ASN 579A)]};
		metallocompound **12** {[HB: 2.59 H(COOH) O(CYS 531A)], [HB: 1.66 O(COOH) H(CYS 531A)]}
	Molegro-Constrained	metallocompound **1** {[HB: 2.04 N(arene) H(SER 535A)], [MR: 4.61 SG(CYS 531 A)]}
		metallocompound **8** {[MR: 4.61 SG(CYS 531 A)]}
	Autodock	metallocompound **4a** {[HB: 1.91 H(NH_2_) ^i^ OD2(ASP 478A)], [HB: 2.03 H(H_2_O) OD2(ASP 478A)], [HB: 1.84 H(NH_2_) ^ii^ OE2(GLU 532A)], [HB: 1.94 H(H_2_O) OE2(GLU 532A)]};
		metallocompound **5a** {[none]}
rHA	Molegro	metallocompound **10** {[HB: 2.24 O(COOH)^i^ H(ARG 117A)], [HB: 2.05 O(COOH)^ii^ H(ARG 186A)]};
		metallocompound **12** {[HB: 2.16 H(COOH) O(ASP 108A)], [MR: 4.27 O(SER 193A)]}
	Molegro-Constrain	metallocompound **10a** {[HB: 2.12 H(COOH) ^i^ OE2(GLU 37A)], [HB: 1.68 O(COOH) ^ii^ H(ARG 144A)], [HB: 1.82 H(COOH) ^ii^ O(GLN 33A)], [MR: 4.59 NH1(ARG 144A)]}
		metallocompound **2** {[HB: 1.91 O(COO)^i^ H(ARG 144A)], [HB: 1.91 O(COO) ^i^ H(ARG 144A)], [MR: 4.59 NH1(ARG 144A)]};
	Autodock	metallocompound **10** {[MR: 1.78 NZ(LYS 195A)], [MR: 4.52 NH_2_(ARG 222A)]};
		metallocompound **4a** {[HB: 1.85 H(H_2_O) OD2(ASP 38A)], [HB: 1.73 H(H_2_O) OH(TYR 84A)], [HB: 1.85 H(NH_2_) ^i^ OD2(ASP 34A)]}
RNR	Molegro	metallocompound **1a** {[HB: 1.59 H(H_2_O) O(PRO 621A)], [HB: 1.98 H(COOH) OE1(GLU 441A)], [HB: 2.39 O(COOH) HD22(ASN 437A)]};
		metallocompound **3** {[HB: 1.88 O(COO) ^i^ H(GLU 623A)], [HB: 1.92 O(COO) ^i^ H(SER 625A)], [HB: 2.34 O(COO) ^ii^ H(THR 209A)]}
	Molegro-Constrained	metallocompound **1a** {[HB: 2.22 H(COOH) O(SER 224B)], [MR: 4.17 SG(CYS 439A)]};
		metallocompound **10** {[HB: 2.27 H(COOH) ^i^ O(PRO 621A)], [HB: 2.16 H(COOH) ^ii^ O(SER 224A)], [MR: 4.26 S(CYS 439A)]}
	Autodock	metallocompound **1a** {[HB: 1.68 H(COOH) OE1(GLU 623A)], [HB: 2.00 O(H_2_O) H(ARG 639A)], [MR: 3.69 NH_2_(ARG 639A)]};
		metallocompound **12** {[HB: 2.60 O(COOH) H(GLU 623A)], [HB: 1.95 O(COOH) N(SER 625A)], [HB: 2.02 O(COOH) HG(SER 625A)], [HB: 1.77 H(COOH) OG1(THR 209A)]}
TopII	Molegro	metallocompound **1** {[HB: 2.15 H(COOH) O(GLN 365B)], [HB: 3.38 O(COOH) O(THR 27A)]}
		metallocompound **10** {none};
	Molegro-Constrained	metallocompound **10a** {[HB: 2.47 H(COOH) O(ILE 15A)], [MR: 4.60 NE2(HIS 20B)]};
		metallocompound **1a** {[MR: 4.60 NE2(HIS 20B)]}
	Autodock	metallocompound **5** {[HB: 2.21 O(COO) H(GLY)], [HB: 2.57 O(COO) NH_2_(GLN 365A)], [HB: 2.53 O(COO) NH_3_(LYS 367A)], [HB: 2.09 O(COO) H(ASN 142A)], [HB: 2.37 O(COO) H(ARG 141A)]};
		metallocompound **9a** {[HB: 1.90 H(COOH) ^i^ OD1(ASN 70A)], [HB: 1.88 O(COOH) ^i^ HZ1(LYS 147A)], [HB: 1.85 H(COOH) ^ii^ OD1(ASN 129A)], [HB: 2.15 O(COOH) ^ii^ HD21(ASN 129A)]}
TrXR	Molegro	metallocompound **10** {[HB: 1.77 O(COOH) ^i^ O(ALA 198A)], [HB: 2.60 O(COOH) ^ii^H(ARG 166A)], [HB: 2.19 O(COOH) ^ii^ H(ARG 166A)]};
		metallocompound **1** {[HB: 2.14 H(COOH) O(SER 222A)], [HB: 1.75 O(COOH) OH(SER 222A)]}
	Molegro-Constrained	metallocompound **9a** {[HB: 2.27 O(COOH)^i^ HZ1(LYS 67A)], [HB: 2.10 H(COOH) ^ii^ O(THR 373A)], [MR: 3.59 SG(CYS 64A)], [MR: 4.60 SG(CYS 59A)]}
		metallocompound **8a** {[HB: 1.63 H(COOH) OD2(ASP 334A)], [HB: 2.11 O(COOH) HH21(ARG 293A)], [HB: 1.85 O(COOH) HE(ARG 293A)], [MR: 3.06 SG(CYS 64A)], [MR: 4.60 SG(CYS 59A)]}
	Autodock	metallocompound **5a** {[HB: 1.96 H(NH_2_) O(GLU 341A)], [HB: 1.89 H(NH_2_) OH(TYR 200A)], [HB: 2.10 O(COO) H(THR 343A)], [HB: 2.13 O(COO) H(THR 343A)], [HB: 2.02 H(H_2_O) OD1(ASP 334A)], [HB: 2.48 H(H_2_O) OD2(ASP 334A)]}
		metallocompound **12** {[HB: 1.69 H(COOH) O(GLU 341A)], [HB: 1.98 O(COOH) NH_3_(LYS 315A)], [MR: 3.47 H(LEU 340A)]};
TS	Molegro	metallocompound **12** {[HB: 2.22 H(COOH) O(SER 232A)]};
		metallocompound **10** {[HB: 1.53 O(COOH) H(ASN 229A)]}
	Molegro-Constrained	metallocompound **10a** {[HB: 2.40 O(COOH) H(CYS 198A)], [HB: 1.86 O(COOH) HH(TYR 146A)], [MR: 4.60 S(CYS 198A)]}
		metallocompound **1** {[HB: 2.13 H(COOH) O(SER 219A)], [HB: 2.70 O(COOH) H(ARG 218A)], [HB: 2.39 O(COOH) H(ARG 218A)], [HB: 2.38 O(COOH) H(ARG 23A)], [HB: 1.56 O(COOH) H(ARG 23A)], [MR: 3.55 S(CYS 198A)]};
	Autodock	metallocompound **9a** {[HB: 1.82 H(COOH) ^i^ O(SER 219A)], [HB: 1.97 O(COOH) ^i^ H(ASP 221A)], [HB: 2.45 O(COOH) ^i^ HG(CYS 198A)], [HB: 2.08 O(COOH) ^ii^ HD21(ASN 229A)], [HB: 1.63 H(COOH) ^ii^ OE2(GLU 60A)]};
		metallocompound **5a** {[HB: 2.36 O(COO) HH(TYR 146A)], [HB: 1.71 H(H_2_O) OE1(GLU 60A)]}

### 2.3. The Binding Affinity of the Metallocompounds to DNA

An insight into the interaction of the metallocompounds with the two types of DNA (DNA-1 and DNA-2) is given for the Autodock (Table 2 and Table 3), relaxed Molegro (Table 4 and Table 5) and constrained Molegro (Table 6 and Table 7) models. There is improved in the binding of many of the complexes to DNA-1 and DNA-2 due to their hydration. Typical examples are complexes **1a**, **8a**, **9a**, **10a** and all the hydrated RAPTA complexes. In agreement with the literature, the best target of cisplatin is DNA [3,28] as the binding affinity decreases significantly in protein receptors compared to DNA-1 and DNA-2. The features of cisplatin in all the dockings shows that its activity significantly increases when it is doubly hydrated (Cisp-W2) than when it is singly hydrated (Cisp-W1). The newly proposed model of complexes appears to have strong DNA interactions that suggest DNA as part of their possible targets. Most of the RAPTA complexes will target CatB or HDAC7 preferentially to DNA. The spectrum of the interactions of RAPTA complexes with DNA range from −116.96 to −49.53, with CatB from −124.88 to −60.05 and with HDAC7 from −127.16 to −55.13 considering both the realaxed and constrained docking which further confirm thats RAPTA complexes will target proteins preferably to DNA [14,15].

**Table 2:** The binding energy of the metallocompounds from the Autodock docking.

	DNA-1	DNA-2	CatB	DNA_Gyrase	HDAC7	HP-NCP	BRAF KINASE	rHA	RNR	topoII	TrxR	TS
**1**	−4.32	−3.3	−4.26	−3.68	−3.15	−3.44	−3.25	−3.45	−3.15	−3	−3.43	−3.03
**2**	−2.47	−3.08	−3.38	−3.21	−2.64	−3.43	−3.79	−4.19	−3.71	−2.65	−3.35	−4.14
**3**	−2.73	−2.5	−3.43	−3.49	−2.5	−3.82	−3.29	−3.91	−3.49	−3.84	−3.35	−3.7
**4**	−4.67	−3.88	−3.98	−3.49	−3.92	−4.16	−3.65	−3.86	−2.85	−3.05	−3.21	−2.84
**5**	−2.93	−2.7	−3.27	−3.01	−3.02	−3.54	−3.39	−3.62	−3.07	−4.08	−3.39	−3.21
**6**	−2.29	−1.9	−1.24	−1.31	−1.77	−2.46	−2.37	−2.32	−2.45	−1.62	−2.19	−1.31
**7**	−2.82	−2.79	−3.42	−3.38	−3.1	−2.59	−3.97	−3.97	−3.6	−3.47	−2.79	−2.96
**8**	−2.24	−3.41	−2.92	−3.09	−2.59	−3.29	−3.39	−3.4	−3.25	−3.03	−3.43	−2.53
**9**	−4.27	−3.73	−4.02	−3.24	−3.5	−3.63	−3.66	−3.34	−3.39	−3.1	−3.92	−3.67
**10**	−5.13	−4.94	−5.19	−4.86	−3.78	−5.14	−4.48	−5.24	−4.03	−3.86	−4.24	−4.51
**11**	−5.02	−3.81	−4.5	−3.68	−4.01	−3.85	−3.12	−2.98	−2.43	−2.19	−2.91	−3.65
**12**	−5.97	−4.65	−5.77	−5.12	−5.1	−4.56	−4.37	−4.16	−4.19	−3.4	−4.31	−5.23
**13**	−2.5	−2.44	−3.35	−2.94	−2.78	−2.84	−3.13	−3.38	−2.88	−2.88	−2.86	−2.78
**14**	−3.04	−3.14	−3.55	−3.54	−2.72	−3.83	−3.41	−4.29	−3.52	−3.98	−3.79	−3.7
**15**	−2.43	−2.56	−3.38	−3.1	−2.82	−3.05	−3.27	−3.39	−3.09	−3.13	−2.83	−2.96
**16**	−1.63	−1.87	−2.23	−2.15	−1.66	−2.22	−2.08	−2.59	−2.41	−2.08	−2.11	−2.4
**cisplatin**	−2.23	−2.94	−2.15	−1.34	−1.98	−2.18	−2.56	−1.65	−1.93	−1.95	−2.4	−1.23
**co-crystallized compounds**			−5.49	−8.68	−4.35				−7.01	−12.48	−4.79	−9.19

**Table 3:** The binding energy of the metallocompounds from the Autodock docking of hydrated complexes.

	DNA-1	DNA-2	CatB	DNA_Gyrase	HDAC7	HP-NCP	BRAF KINASE	rHA	RNR	topoII	TrxR	TS
**1a**	−6.53	−5.65	−6.47	−4.67	−5.97	−4.95	−4.97	−4.04	−4.39	−3.16	−4.26	−4.9
**4a**	−6.85	−5.87	−6.29	−4.82	−5.55	−5.58	−5.27	−4.54	−3.16	−3.47	−4.23	−4.72
**5a**	−5.58	−4.75	−5.65	−4.55	−5.08	−5.26	−4.98	−4.33	−4.13	−3.96		−5.35
**6a**	−5.35	−3.6	−3.6	−2.3	−3.39	−4.51	−4.26	−2.6	−2.37	−2.38	−3.54	−2.96
**8a**	−6.44	−5.03	−5.78	−4.76	−5.25	−5.22	−3.96	−4.02	−3.02	−3.06	−2.98	−4.2
**9a**	−6.98	−5.96	−6.19	−5.03	−5.56	−5.05	−4.77	−3.3	−3.95	−4.07	−3.94	−6.09
**10a**	−6.38	−5.22	−6.7	−4.61	−5.21	−4.58	−4.03	−4.48	−3.49	−3.51	−4.17	−5.26
**13a**	−4.86	−4.06	−5.19	−4.05	−4.81	−4.29	−3.59	−3.74	−3.32	−3.65	−3.61	−3.39
**14a**	−4.39	−3.75	−4.84	−4.19	−4.59	−4.32	−3.33	−3.99	−3.65	−3.73	−3.76	−3.17
**15a**	−4.86	−4.03	−4.38	−3.8	−4.79	−4.27	−3.64	−3.41	−3.14	−3.6	−3.65	−3.05
**16a**	−4.12	−3.24	−3.1	−3.28	−3.75	−3.62	−3.1	−2.91	−2.16	−2.77	−2.65	−2.31
**Cisp-W1**	−4.31	−3.98	−2.1	−0.99	−2.55	−3.37	−3.25	−1.73	−1.04	−1.15	−2.63	−1.45
**Cisp-W2**	−4.92	−4.4	−2.15	−0.93	−2.76	−3.18	−3.47	−1.72	0.02	−0.33	−1.79	−1.2

**Table 4:** The binding energy of the metallocompounds from the relaxed Molgro docking.

	DNA-1	DNA-2	CatB	Gyrase	HDAC7	HP-NCP	BRAF Kinase	rHA	RNR	TopII	TrXR	TS
**1**	−137.57	−133.77	−136.37	−131.99	−166.09	−111.45	−118.79	−142.13	−126.87	−154.82	−137.87	−127.39
**2**	−113.84	−106.15	−124.88	−110.82	−127.16	−94.13	−110.31	−113.18	−98.90	−121.50	−103.71	−114.61
**3**	−116.96	−97.09	−114.43	−103.00	−110.06	−91.82	−105.79	−135.47	−124.93	−118.69	−104.43	−111.20
**4**	−77.36	−77.97	−72.59	−79.89	−83.51	−68.21	−67.34	−85.02	−74.88	−74.75	−75.04	−75.89
**5**	−71.22	−82.12	−78.61	−73.09	−90.05	−68.85	−74.96	−86.25	−83.41	−84.15	−82.63	−79.76
**6**	−86.14	−81.39	−88.78	−84.63	−95.90	−75.53	−84.76	−97.60	−88.56	−92.57	−100.22	−79.41
**7**	−87.95	−84.94	−93.69	−94.11	−102.98	−75.06	−99.78	−100.28	−92.07	−97.69	−106.18	−83.43
**8**	−119.77	−111.18	−118.67	−110.79	−107.93	−96.84	−117.76	−126.06	−116.02	−115.67	−121.37	−111.59
**9**	−124.03	−122.96	−125.25	−126.26	−131.56	−101.43	−115.00	−119.84	−106.90	−129.34	−119.82	−118.59
**10**	−141.56	−126.74	−135.89	−142.43	−147.55	−107.85	−114.94	−147.15	−121.72	−129.66	−138.35	−129.75
**11**	−143.11	−142.45	−131.53	−140.36	−157.61	−117.62	−114.61	−133.71	−114.68	−128.89	−121.04	−127.35
**12**	−153.94	−129.90	−144.21	−138.83	−160.63	−104.74	−122.12	−142.37	−113.66	−129.13	−120.89	−135.47
**13**	−62.98	−67.16	−83.32	−70.45	−90.53	−63.02	−73.54	−73.40	−74.49	−82.38	−70.73	−77.41
**14**	−82.56	−85.26	−100.23	−85.99	−113.53	−71.41	−89.94	−97.88	−91.04	−102.67	−89.64	−85.02
**15**	−66.45	−67.86	−89.21	−70.59	−101.13	−62.61	−74.58	−78.25	−81.13	−84.18	−78.17	−84.17
**16**	−73.52	−76.16	−95.70	−80.20	−94.92	−71.53	−87.20	−87.77	−80.40	−93.80	−84.48	−87.89
**Cisplatin**	−47.86	−44.53	−38.09	−46.03	−45.22	−46.40	−39.91	−43.53	−40.83	−43.89	−38.82	−39.79
**co-crystallized compounds**			−117.36	−162.48	−70.17		−121.74		−152.80	−192.15	−160.24	−106.57

**Table 5:** The binding energy of the metallocompounds from the relaxed Molgro docking for hydrated compounds.

	DNA-1	DNA-2	CatB	Gyrase	HDAC7	HP-NCP	BRAF Kinase	rHA	RNR	TopII	TrXR	TS
**1a**	−150.05	−145.76	−128.02	−136.31	−106.38	−129.59	−122.79	−137.56	−125.01	−84.25	−131.96	−127.11
**4a**	−94.36	−98.02	−77.65	−81.91	−81.50	−76.86	−61.37	−80.77	−73.66	−75.59	−87.09	−79.70
**5a**	−88.43	−96.33	−82.01	−76.01	−86.05	−75.74	−68.95	−87.31	−80.07	−82.79	−87.95	−86.37
**6a**	−100.04	−95.69	−91.02	−89.94	−96.65	−73.28	−85.05	−104.25	−86.02	−81.42	−99.80	−82.86
**8a**	−146.26	−127.46	−126.15	−136.28	−129.26	−113.66	−113.13	−129.33	−113.82	−116.42	−111.76	−118.85
**9a**	−144.54	−125.87	−123.34	−120.42	−90.58	−106.68	−109.38	−127.80	−106.99	−44.91	−131.76	−120.86
**10a**	−161.33	−145.84	−131.71	−142.22	−158.93	−113.82	−110.56	−121.54	−120.44	−79.33	−122.11	−126.24
**13a**	−77.52	−81.55	−90.06	−75.72	−94.57	−71.10	−71.20	−74.88	−80.32	−88.55	−71.37	−79.81
**14a**	−96.88	−106.30	−105.80	−99.03	−110.38	−80.03	−95.53	−86.14	−94.44	−106.7	−94.05	−86.26
**15a**	−84.66	−86.84	−91.02	−82.81	−101.74	−74.49	−74.40	−78.79	−83.59	−95.58	−80	−75.33
**16a**	−89.62	−89.80	−98.32	−85.32	−106.94	−81.19	−85.06	−90.37	−82.83	−99.96	−81.1	−84.77
**Cisp-W1**	−62.38	−58.09	−46.27	−50.24	−44.07	−44.94	−35.32	−44.08	−41.17	−42.72	−44.55	−42.56
**Cisp-W2**	−84.39	−70.95	−45.17	−57.47	−43.45	−42.73	−28.77	−37.91	−43.82	−42.74	−44.54	−41.68

**Table 6:** The binding affinities of the metallocompounds from the covalent constrained Molegro docking.

	DNA-1	DNA-2	CatB	Gyrase	HDAC7	HP-NCP	BRAF Kinase	rHA	RNR	TopII	TrXR	TS
**1**	−116.11	−128.23	−99.45	−107.80	−133.48	−95.08	−105.04	−27.44	−121.13	−43.60	−67.44	−110.86
**2**	−97.80	−85.91	−82.34	−84.02	−74.26	−72.24	−10.28	−86.36	−99.26	−8.38	−14.82	−98.92
**3**	−78.05	−72.06	−74.09	−72.94	−66.77	−76.14	−58.45	10.16	−90.08	−72.78	−40.92	−81.43
**4**	−68.50	−61.59	−67.53	−50.22	−71.28	−61.87	−63.35	−43.68	−60.08	−38.15	−39.06	−54.89
**5**	−61.04	−58.83	−63.33	−57.28	−76.44	−58.64	−70.82	26.31	−65.84	−44.97	−41.88	−61.54
**6**	−71.93	−66.06	−72.19	−62.16	−94.46	−61.01	−65.64	−70.29	−71.03	−43.94	−47.54	−69.91
**7**	−70.49	−65.18	−75.95	−67.76	−96.59	−67.18	−69.16	15.13	−75.00	−63.31	−53.38	−70.06
**8**	−90.84	−88.44	−79.93	−82.66	−83.25	−81.53	−96.36	−2.29	−96.45	−49.40	−63.45	−102.19
**9**	−111.80	−117.06	−99.06	−110.65	−87.96	−78.92	−79.57	−49.97	−104.49	−70.13	−62.38	−92.44
**10**	−122.46	−117.40	−90.07	−90.46	−141.61	−102.69	−74.46	−88.52	−113.02	−47.87	−45.13	−99.57
**11**	−124.47	−125.50	−111.86	−104.70	−144.86	−104.53	−92.96	−85.74	−107.52	−50.71	−91.01	−98.64
**12**	−125.54	−129.90	−104.35	−107.26	−112.43	−97.12	−75.51	−66.29	−106.43	−8.28	−66.59	−91.48
**13**	−49.78	−49.53	−60.05	−48.08	−55.13	−46.22	−59.27	−47.94	−62.99	−56.55	−28.91	−58.53
**14**	−66.71	−68.14	−79.05	−65.58	−66.86	−51.48	−50.64	19.86	−77.96	0.00	−52.96	−78.21
**15**	−54.61	−55.65	−64.40	−58.80	−58.83	−53.29	−63.23	−55.84	−64.54	−57.33	−36.73	−65.11
**16**	−65.29	−64.79	−70.31	−68.07	−72.82	−58.20	−58.63	−66.45	−73.20	−37.72	−35.54	−71.26
**Cisplatin**	−38.97	−34.23	−43.49	−33.31	−49.14	−29.06	−36.20	−26.49	−30.79	−35.58	−33.00	−30.63
**co-crystallized compounds**			−132.12	−120.11	−73.24	−30.80	−94.68		−126.37	−115.68	−144.33	−95.40

**Table 7:** The binding affinities of the metallocompounds from the covalent constrained Molegro docking of hydrated complexes.

	DNA-1	DNA-2	CatB	Gyrase	HDAC7	HP-NCP	BRAF Kinase	rHA	RNR	TopII	TrXR	TS
**1a**	−126.70	−122.83	−127.33	−117.35	−99.20	−104.06	−86.78	−7.23	−113.75	−81.83	−101.44	−108.38
**4a**	−85.71	−72.54	−69.40	−65.33	−81.49	29.93	−59.98	36.01	−59.88	−39.37	−63.58	−53.49
**5a**	−77.64	−71.78	−68.57	−63.59	−86.11	−57.75	−67.38	29.87	−63.13	−47.71	−67.64	−64.37
**6a**	−94.75	−84.34	−81.49	−74.57	−96.64	−66.72	−64.99	24.66	−76.07	−54.41	−69.31	−73.20
**8a**	−128.21	−111.69	−110.41	−112.41	−129.27	−99.85	−92.82	−78.11	−110.43	−69.52	−103.76	−94.83
**9a**	−134.05	−125.46	−103.79	−119.20	−65.16	−81.50	−74.88	−52.63	−106.48	−68.35	−118.89	−100.43
**10a**	−135.60	−126.76	−116.86	−120.84	−158.92	−115.24	−72.50	−91.28	−110.44	−82.45	−84.59	−113.85
**13a**	−63.52	−63.46	−69.71	−57.52	−73.39	−60.84	−58.70	35.82	−67.58	−59.59	−33.43	−55.79
**14a**	−84.65	−78.01	−92.77	−69.23	−74.84	−60.37	−54.22	23.16	−82.70	−5.71	−11.37	−78.60
**15a**	−71.57	−68.76	−75.77	−66.29	−72.91	−66.99	−64.27	−37.41	−72.72	−66.08	−36.32	−62.97
**16a**	−74.70	−74.07	−73.81	−74.55	−72.88	−69.08	−60.26	−34.70	−76.95	−46.59	−42.19	−66.71
**Cisp-W1**	−62.37	−45.20	−40.91	−36.93	−43.65	−35.62	−32.23	−31.06	−35.91	−29.93	−34.13	−29.65
**Cisp-W2**	−78.38	−55.98	−34.03	−38.27	−43.46	−40.96	−29.51	−24.29	−32.68	−25.48	−32.34	−26.43

### 2.4. The Binding Affinities of the Metallocompounds to CatB and TrxR

The results obtained from relaxed Molegro and Autodock docking (Table 2, Table 3, Table 4 and Table 5) show that **12**, **1** and **1a**, **10** and **10a**, **9** and **9a** and **11** bind strongly to CatB than its co-crystallized compound. Both Molegro and Autodock included the hydrated RAPTA complexes as part of the best binding metallocompounds to CatB while only Molegro ranked bidentate RAPTA complexes **2** and **3** better than the hydrated ones. In ranking, we give preference to the Molegro dockings than the Autodock ones, especially where there is a very close binding affinity prediction from the Autodock docking considering the standard error margin of ~2.177 kcal/mol [25,26] which is an indication that metallocompounds within this range of binding can be reordered. In the binding affinities of the metallocompounds to TrXR, the new models are still rated best followed by the bidentate RAPTA complexes (compounds **3** and **2**) and the hydrated RAPTA complexes. The features of the predicted binding affinities of RAPTA complexes give a better insight into their reported experimental activities in CatB and TrXR. The experimental activities of the RAPTA complexes against CatB follow the order **15**, **14**, **2**, while **3** and **13** rarely show any appreciable binding affinity and that of TrXR followed the order **2**, **3**, **14** and **15** while **13** has the lowest activity (Table 8) [14]. Selecting only the metallocompounds that have experimental values, the predicted binding affinities of the metallocompounds against TrXR using Molegro agree better with the experimental order of their inhibitory activities as metallocompound **13** is rated least in activities toward TrXR followed by metallocompound **15**. Contrary to the experimental report, metallocompound **3** was predicted to bind strongly to CatB. However, the ranking of metallocompounds **14a** and **15a** among those that have the best binding to CatB and metallocompound **13** rated least agrees well with the experiment (Table 8). The rating of both hydrated and non-hydrated forms of metallocompound **14a** among those that bind strongly to CatB, further give insight into their experimental finding as the best anticancer agents among the RAPTA complexes [5]. The three docking methods show that the interaction of the metallocompounds with CatB is stronger than with TrXR, which agrees well with the experimental report [14]. The binding affinities of the hydrated metallocompounds with CatB and TrXR are significantly enhanced (Table 2, Table 3, Table 4, Table 5, Table 6 and Table 7)

### 2.5. The Binding Affinity of the Metallocompounds with Gyrase, HDAC7, HP-NCP, BRAF Kinase, rHA, RNR, TopII and TS

The features of the binding affinities of the metallocompounds against gyrase from both docking methods show that co-crystallized compound still preferentially bind to gyrase better than any of the metallocompounds, even though the activities of the new modeled metallocompounds are competitively close to that of its co-crystallized compound. The metallocompounds that have the best binding to gyrase using Molegro are **1** and **1a**, **9** and **9a**, **10** and **10a**, **11** and **12** (Table 4, Table 5, Table 6 and Table 7). Autodock included the hydrated RAPTA complexes **14a**, **13a** and **15a** immediately after metallocompounds **12**, **9a**, **10**, **4a**, **8a**, **1a**, **10a** and **5a** (Table 2 and Table 3). The binding affinities of the metallocompounds to HDAC7 show that the models **1**, **12**, **11**, **10**, **9** and their respective hydrolytic forms (where applicable) have better binding than its co-crystallized compound. This is also applicable to the RAPTA complexes.

**Table 8:** The correlation of the factors that determine the biding interaction of the metallocompounds with the receptors using Molgro docking (unconstrained docking).

CatB				
	Experiment	Moldock-non	Moldock-cons	Autodock
RAPTATh2o	1.5	−91.0243	−75.7655	−4.38
RAPTACh2o	2.5	−105.798	−92.7676	−4.84
CRAPTAC	5	−124.877	−82.3353	−3.38
ORAPTAC	200	−114.427	−74.0878	−3.43
RAPTABh2o	200	−90.0599	−69.7078	−5.19
**TrXR**				
CRAPTAC	4.6	−103.714	−14.8246	−3.35
ORAPTAC	32.5	−104.425	−40.9218	−3.35
RAPTACh2o	37.1	−94.0454	−52.9618	−3.76
RAPTATh2o	144	−80.0042	−36.727	−3.65
RAPTABh2o	200	−71.3701	−28.9068	−3.61

There have been experimental reports on HP-NCP also being a possible target of RAPTA complexes [15], but the new models metallocompounds **1**, **9**, **10**, **11**, **12** and their hydrated forms (where applicable) are predicted to bind better than RAPTA complexes by all three methods of docking. The crystal structure of HP-NCP shows RAPTA-C (metallocompound **14a**) binding to two different sites on chain G and H of the receptor (Figure 2e). In order to make a parallel comparison of the poses of metallocompound **14a** with the available experimental crystal structure, its docking features are shown in Figure 2f. The constrained Molegro and Autodock docking of complex **14a** gives preference to the first binding site, which suggests that complex 14a will bind preferentially to the first site than the second. Also, the constrained Molegro docking shows that the PTA unit of this metallocompound locates the same receptor pocket as the crystal structure (Figure 2e). The new models of metallocompounds **1**, **9**, **10**, **11**, **12** and **8** (include their hydrated forms) are predicted among those that have the best binding to BRAF kinase (Table 4 and Table 5). BRAF kinase has been proposed as a target of the “piano stool” type of Ru complexes [16]. This “piano stool” which was treated as a co-crystallized compound during docking was predicted in the relaxed Molegro docking (Table 4 and Table 5) to have the affinity closest to the best binding complexes **1a** and **12**. The best binding metallocompounds to rHA from the three methods of docking are still **10**, **12**, **1**, **3**, **11**, **8**, **9**, **3** and **14** (including their hydrated forms where applicable). The rHA is predicted as an average target, suggesting that many of the Ru(II)-based complexes that are considered will on average be kinetically favourable which further support the experimental findings [3,5,27].

In the interaction of the metallocompounds with RNR, the binding site co-crystallized compound is found to bind preferentially to all the metallocompounds. The predicted best binding metallocompounds to RNR are **1**, **10**, **8**, **11**, **12** and **9** including the bidentate RAPTA complexes **3**, **2** and hydrated **14a**. The suggested best binding metallocompounds to TopII after the co-crystallized compound are **1**, **10**, **9**, **12**, **11** and the RAPTA complexes **2**, **3**, **14a** and **16a**. Commonly predicted best binding metallocompounds to TS by the three methods of docking are **12**, **10**, **11**, **9** and the bidentate RAPTA complexes **2** and **3** while only Molegro rated **1** as part of the best for TS.

### 2.6. Metal-Receptor Residues Covalent Interaction Using Constrained Molegro Docking

Since the targets of many organometallic complexes are not yet known [5,6,7,8,9], there is yet to be clear evidence of their metal centre forming covalent bonding with the residues of the receptors. In docking, the possible covalent interaction of the metal with any of the target residues can best be computed using constraints [14] but the application is usually limited to the systems in which such interactions have been experimentally proven. Generally, it is understood that the metal centre (the acidic centre) in metallocompounds may interact with nucleophilic centres (the basic centres) like the sulphur atom of the thiol in cysteine and the thioether in methionine, the imidazole nitrogen atom in histidine (*i.e*., for protein receptors) and the N7 atom of the guanine in DNA [11,30]. There have been both reports of inhibitory activities of metal complexes where there is no observed metal-residues covalent interaction even within the 4.00 Å distance from the metal centre [16,31] and in cases in which there is the possibility of a covalent bond between the metal centre of metallocompounds and the receptor residues [15]. There is a clear indication that the metal atoms in many cases prefer to hold the ligands together for synergistic effects [17] and for better interaction with the receptor residues than forming covalent bonds. In order to apply the possibility of covalent bonding of the metal centre with the receptor residues, we apply a distance constraint of 2.00 to 4.60 between the metal and the nucleophilic center of residues that makes up the receptor-binding site. In this study, based on the available information on the binding site residues of each receptor, a nucleophilic atom was selected as centre of the interaction with the metal centre of the metallocompounds. The cysteine sulphur atom (SG) was selected for CatB (Cys A 29) [32], BRAF kinase (CYS A 531) [16], RNR (CYS B 439) [33], TrXR (CYS A 59) [34] and TS (CYS A 198) [35]; a histidine nitrogen atom was used for HDAC7 (HIS A 709) [36], HP-NCP (HIS H 106) [15] and TopII (HIS B 20) [37]; while for gyrase [23] and rHA [31] where none of the HIS, CYS and MET are within the binding site residues, the nucleophilic nitrogen atoms of ASN A 46 and ARG A 144 were used, respectively. For DNA (4DL7) [38], the nitrogen atom (N7) of the guanine (DG T 7) was used.

The general feature is that the binding affinity is lower when the covalent constraint is applied (Table 6 and Table 7) compare to when it is not (Table 4 and Table 5). This therefore shows that the ruthenium metal in many of the metallocompounds will prefer to position the ligands for optimum receptor interactions than to form covalent bonds with the residues of protein receptors. However, the binding affinities of some metallocomponds against targets like DNA-1, DNA-2, HDAC7, HIS and RNR during the covalent constrained docking is comparatively high as that of the relaxed docking, indicating the possibility of the ruthenium atom forming covalent interactions with the receptor residues. Whether the constraint is applied or not, some of our model metallocompounds like **1**, **9**, **10**, **11** and **12** (including their hydrated forms where applicable) are still ranked among those that have the best binding affinities to all the receptors. The binding behaviour of many of the metallocompounds when the covalent constraint is applied or omitted shows that the functions of the metal centre of the metallocompounds are receptor dependent.

In summary, the new models, especially complexes **1**, **9**, **10** and **12** (and their respective hydrated forms where applicable) are predicted by all the methods of docking between the best two inhibitors of all the receptors. Also, complex **11** is rated among the best three during the relaxed docking for gyrase and during the constrained docking for CatB and HDAC7. Specific to Autodock docking, the hydrated complexes **4a** and **5a** are suggested among the best inhibitors of DNA-1, DNA-2, HP-NCP, Kinase, TopII, TrXR and TS. Ranked high among the RAPTA complexes in many of the receptor interactions are the bidentate complexes **3** and **4** followed by the hydrated complexes **14a** and **16a**. This gives further insight into the reported better anticancer activities of complexes **14a** [5] and **16a** [27]. Considering the ranking of the three methods of docking, the best five metallocompounds predicted as inhibitors of the receptors are mostly within the proposed new models except in TopII, RNR and rHA where some of the RAPTA complexes are ranked among the best five. However, there is none of the RAPTA complexes appear among the best two proposed inhibitors of the receptors considered in this work using the three methods of docking (Table 1) except in rHA and RNR, where the ranked first and the second complexes are the non-hydrated and hydrated form of the same compound (Table 4, Table 5, Table 6 and Table 7). This suggests the possibility of the new models acting a better anticancer than the RAPTA complexes.

### 2.7. The Correlation of Factors that Determined the Binding Activities of the Metallocompounds

The docking method of Molegro gives a more comprehensive insight into the factors that determine the binding affinities of the metallocompounds. Their correlation Table 8 and Table 9 are constructed using the statistical package called R [39]. The most significant factors that influence the metallocompounds interaction with the DNA and many of the protein receptors are intramolecular van der Waal (E.Intra.vdw.), pose energy, steric and long range electrostatic interaction (ElectroLong in Table 8 and Table 9). In all the protein receptors, the inter protein-ligand and the pose energies significantly determine the total binding energy of the metallocompounds. The presence of higher number of heavy atoms is proposed to favour better binding energy in all the receptor-ligand interaction. The presence of halogens also averagely favours the ligand-receptor interaction. A higher torsional bonds and lower steric hindrance are shown to favour the binding interaction of the metallocompounds. In the unconstrained docking, the short electrostatic interaction (Electro in Table 8) plays significant role in the binding affinity of the metal complexes to the receptors gyrase and RNR and the long range electrostatic interactions (ElectroLong in Table 8) influence the binding to DNA, TrXR and TS. The HB strongly affects the metallocompounds binding to TrXR while Van der Waal forces strongly influence metallocompounds binding to HP-NCP (VdW in Table 8 and Table 9). In the constrained system, soft constraints strongly influence the binding affinities of metallocompounds to BRAF kinase and rHA. Also, steric interaction energies (Steric in Table 8 and Table 9) between the receptors and the metallocompounds strongly influence the binding affinities in both constrained and unconstrained docking.

**Table 8:** The correlation of the factors that determine the biding interaction of the metallocompounds with the receptors using Molgro docking (unconstrained docking).

	4DL7	CatB	Gyrase	HDAC7	HP-NCP	BRAF Kinase	rHA	RNR	TopII	TrXR	TS
E.Inter.protein.ligand.	0.28	0.91	0.93	0.93	0.89	0.89	0.93	0.92	0.81	0.90	0.90
E.Intertotal	0.84	0.91	0.93	0.93	0.89	0.89	0.93	0.92	0.81	0.90	0.90
E.Intra.steric.	0.12	0.42	0.39	0.46	0.68	0.49	0.66	0.58	0.45	0.31	0.55
E.Intra.tors.	0.26	−0.01	−0.11	0.07	0.20	0.05	0.15	−0.11	−0.10	−0.53	0.14
E.Intra.tors.ligandatoms.	0.16	0.37	0.30	0.43	0.65	0.46	0.63	0.51	0.36	−0.01	0.52
E.Intra.vdw.	−0.91	−0.77	−0.82	−0.79	−0.67	−0.65	−0.69	−0.50	−0.32	−0.58	−0.74
Electro	−0.35	0.22	0.45	−0.14	−0.07	−0.04	−0.12	0.61	−0.19	0.26	0.07
ElectroLong	−0.76	0.20	0.31	−0.08	−0.21	−0.06	−0.29	0.36	−0.20	0.53	0.51
HBond	−0.47	0.20	0.17	−0.44	0.03	−0.14	0.11	0.34	0.13	0.71	0.31
HeavyAtoms	−0.71	−0.91	−0.82	−0.84	−0.80	−0.85	−0.76	−0.78	−0.52	−0.82	−0.84
LE1	−0.13	−0.50	−0.20	−0.39	−0.21	−0.39	−0.11	−0.27	−0.23	−0.22	−0.30
LE3	−0.11	−0.63	−0.39	−0.45	−0.43	−0.41	−0.42	−0.27	−0.40	−0.52	−0.11
MW	−0.51	−0.69	−0.65	−0.64	−0.60	−0.67	−0.54	−0.53	−0.13	−0.71	−0.56
N	−0.51	−0.57	−0.35	−0.63	−0.59	−0.49	−0.47	−0.63	−0.60	−0.64	−0.53
NoHBond90	−0.35	0.19	0.15	−0.15	0.08	0.01	0.22	0.64	0.45	0.74	0.31
PoseEnergy	0.80	0.99	0.99	0.99	0.99	0.99	1.00	0.99	0.99	0.99	0.99
RerankScore	0.81	0.69	0.48	0.08	0.52	0.54	0.32	0.85	−0.23	0.38	0.53
Steric	0.48	0.85	0.90	0.94	0.89	0.87	0.92	0.77	0.80	0.85	0.86
Torsions	−0.31	−0.31	−0.55	−0.20	−0.41	−0.45	−0.38	−0.65	−0.27	−0.70	−0.37
VdW.LJ12.6.	0.32	−0.01	−0.17	−0.30	−0.11	0.04	−0.29	0.35	−0.34	−0.37	0.20
halogen	0.26	0.38	0.50	0.29	0.40	0.40	0.45	0.50	0.51	0.50	0.42

**Table 9:** The correlation of the factors that determine the binding interaction of the metallocompounds with the receptors using Molgro docking (constrained docking)

	4DL7	CatB	Gyrase	HDAC7	HP-NCP	BRAF Kinase	rHA	RNR	TopII	TrXR	TS
E.Inter.protein.ligand.	−0.43	0.87	0.87	0.94	0.83	−0.17	0.31	0.89	0.67	0.52	0.85
E.Intertotal	0.82	0.87	0.87	0.94	0.83	−0.17	0.31	0.89	0.67	0.52	0.85
E.Intra.steric.	0.15	0.32	0.25	0.67	0.71	−0.39	0.29	0.53	−0.04	−0.18	0.61
E.Intra.tors.	0.30	0.14	−0.01	0.46	0.35	−0.05	−0.21	−0.21	−0.25	−0.51	−0.04
E.Intra.tors.ligandatoms	0.21	0.31	0.22	0.68	0.70	−0.37	0.22	0.44	−0.09	−0.28	0.54
E.Intra.vdw.	−0.71	−0.55	−0.79	−0.55	−0.65	−0.55	−0.43	−0.58	0.00	−0.71	−0.51
E.SoftConstraintPenalty	0.14	0.15	0.46	−0.01	−0.37	0.76	0.89	NA	0.33	0.41	−0.45
Electro	0.05	0.57	−0.33	−0.24	−0.07	−0.24	−0.10	0.19	0.06	−0.01	0.31
ElectroLong	−0.73	0.18	−0.33	0.16	0.72	0.17	0.08	−0.31	0.16	−0.33	−0.36
HBond	0.04	0.10	0.42	0.12	0.10	0.15	0.25	0.53	0.17	0.62	0.34
HeavyAtoms	−0.53	−0.71	−0.81	−0.47	−0.65	−0.37	−0.71	−0.84	0.15	−0.67	−0.82
LE1	0.08	−0.10	−0.20	0.16	0.06	0.78	0.79	−0.14	0.72	0.40	−0.10
LE3	−0.02	−0.15	−0.25	0.09	0.17	−0.42	−0.59	−0.54	0.45	−0.09	−0.47
MW	−0.39	−0.35	−0.67	−0.23	−0.40	−0.50	−0.75	−0.61	0.39	−0.57	−0.55
N	−0.26	−0.42	−0.38	−0.41	−0.49	−0.18	−0.43	−0.63	−0.08	−0.73	−0.57
NoHBond90	0.36	0.40	0.47	0.30	0.13	0.21	0.24	0.70	0.18	0.70	0.31
PoseEnergy	0.83	0.99	0.99	0.99	0.99	1.00	0.99	0.99	0.99	0.99	0.98
RerankScore	0.07	0.05	0.10	0.25	0.66	−0.40	−0.45	0.34	0.46	−0.03	−0.02
Steric	−0.20	0.81	0.85	0.92	0.72	−0.19	0.25	0.87	0.64	0.54	0.79
Torsions	−0.25	−0.62	−0.55	−0.04	−0.16	−0.17	−0.62	−0.63	−0.17	−0.70	−0.51
VdW.LJ12.6.	−0.11	−0.20	−0.23	0.08	0.48	−0.38	−0.44	−0.36	0.41	−0.17	−0.36
halogen	0.37	0.44	0.38	0.35	0.45	−0.32	0.14	0.44	0.17	0.33	0.38

## 3. Computational Methods

The geometries of the metallocompounds were first optimized in Gaussian 03/09 [40,41] using PBE0 hybrid density functional [42] with two different basis sets of 6-31G* for all atoms order than Ru, Cl and P, which were defined with SBKJC VDZ [43] with effective core potential. The atomic charges were recalculated using b3lyp hybrid functional [44] and all electron minimal basis set 3-21g [45] in order to have a uniform representation of the charges. The atomic charges were incorporated into the docking packages for each of the metallocompounds. The docking activities were done using Autodock 4.2 [46] and Molegro [47] that combines differential evolution with a cavity prediction algorithm. The parameter file of the Autodock was further modified to incorporate ruthenium metal van der Waals and other needed parameters which were obtained from the Autodock website [48] but Molegro has built-in parameters that recognise the ruthenium metal. In Autodock, the number of grid points in x, y, z-axes was set to 60 × 60 × 60 with each point separated by 0.375 Å. Docking calculations were carried out with Lamarkian Genetic Algorithm (LGA). Step sizes of 2Å for translation and 50° for rotation were chosen, the maximum number of energy evaluations was set to 2, 500,000 and for each of the 20 independent runs, a maximum number of 27,000 GA operations were generated on a single population of 100 individuals. In Molegro, the docking scoring function of MolDock which make use of piece-wise linear potential (PLP) was used. The maximum iteration was set to 2,500 against the default 1,500 and the population number also increased from default values of 50 to 100. Five maximum poses were selected and a more stringent re-scoring applied on them for a better prediction of the binding activities of the metallocompounds. MolDock scoring considers the hydrogen bonding, inter molecular protein-ligand and intra molecular ligand interactions and has been successfully applying for molecular docking [49]. Since the Molgro docking package is designed for the ligand-protein interaction, the simple trick employed to consider the ligand-DNA interaction is to include DNA as a cofactor of the protein. This is achieved by incorporating protein into the DNA pdb file 3LPV (named DNA-1) or using pdb 4DL7 input which contain both DNA and protein (named DNA-2) obtained from protein data base [50]. The view of the docking results and analysis of their surface with graphical representations were done using UCSF Chimera package [51].

## 4. Conclusion

In this paper we have presented the binding affinities of new model metallocompounds and some of the experimentally available metallocompounds, especially those of RAPTA complexes using the Molegro and Autodock methods of docking. Making use of the atomic charges of the metallocompounds obtained from the optimized quantum calculation significantly improved the predicted activities and ranking of the metallocompounds, which strongly agrees with the available experimental results. In summary, the new models, especially the complexes **1**, **9**, **10** and **12** (and their respective hydrated versions where applicable) are predicted by all the methods of docking to be between the best two inhibitors of all the receptors. Ranked high among the RAPTA complexes in many of the receptor interactions are the bidentate complexes **3** and **4** followed by the hydrated complexes **14a** and **16a** in agreement with reported better anticancer activities of complexes **14a** [5] and **16a** [27]. Considering the ranking of both relaxed and constrained Molegro docking, the best targets of the metallocompounds are in the order HDAC7 > DNA-1 > rHA > CatB > DNA-2 > Gyrase > TrXR > TopII > TS > RNR > HiS > Kinase except in few cases like complex **12** that prefers CatB to rHA. The general features are that the binding affinity is lower when the covalent constraint is applied (Table 6 and Table 7) compare to when it is not (Table 4 and Table 5). However, the binding affinities of some metallocomponds against targets like DNA-1, DNA-2, HDAC7, HIS and RNR during the covalent constrained docking is competitively as high as that of the relaxed docking, indicating the possibility that the ruthenium atom forms covalent interactions with the receptor residues. Also, only the new model metallocompounds are found to display strong binding affinities against DNA either as cofactor in Molegro or pure DNA as in Autodock docking of the metallocompounds against DNA-1 and DNA-2. The RAPTA complexes are suggested to target proteins like CatB and HDAC7 better than DNA, which further confirms the experimental suggestion that DNA may not be the target of RAPTA complexes [14]. The ranking order of the Molegro agrees better with the available experimental reports on some of the RAPTA complexes than the Autodock one, which is found to be within the error margin of Autodock predictions. The proposed mechanism of activation of metallocompound through hydration [2,28] is supported by the three methods of docking. Many of the hydrated forms of the complexes are found to significantly bind stronger than the non-hydrated complexes in many of the receptors. Also, the binding affinities of both hydrated and non-hydrated metallocompounds **16** and **16a** respectively are higher than that of metallocompounds **15** and **15a** by the Molegro, which further agrees with the experimental reports [27]. Those predicted among the RAPTA complexes to have the best binding to many of the receptors are bidentate and hydrated RAPTA complexes **2**, **3**, **14a**, **15a** and **16a**. The lower binding affinities of the metallocompounds in the constrained metal-residues covalent bond type of docking compare to the relaxed Molegro docking and better correlation of the relaxed Molgro to the available experimental reports show that the metal atom in these type of metallocompounds will give preference to positioning the coordinated ligands in the right position for optimal receptor interactions and synergistic effects on each other than forming a covalent bond. Also from the Molegro docking, there is clear indication that the pose energy, inter protein ligand energy, intra molecular van der Waal and molecular weight strongly determine the final binding energy of the metallocompounds to the receptors.
